# Chiral Polythiophenes: Part I: Syntheses of Monomeric Precursors

**DOI:** 10.3390/molecules26144205

**Published:** 2021-07-10

**Authors:** Dorota Krasowska, Rafał Karpowicz, Józef Drabowicz

**Affiliations:** 1 Centre of Molecular and Macromolecular Studies, Polish Academy of Sciences, Sienkiewicza 112, 90-363 Lodz, Poland; 2 Department of Organic Chemistry, Faculty of Chemistry, University of Łódź, Tamka 12, 91-403 Łódź, Poland; 3 Institute of Chemistry, Jan Dlugosz University in Czestochowa, Armii Krajowej Ave. 13/15, 42-200 Czestochowa, Poland

**Keywords:** chirality, monomers, polythiophenes, synthesis, thiophene functionalizations

## Abstract

The purpose of this mini-review is to comprehensively present the synthetic approaches used for the preparation of non-racemic mono- and multi-substituted thiophenes, which, in turn, can be applied as precursors for the synthesis of chiral polythiophenes isolated as a single chemical entity or having supramolecular thin-layer architectures.

## 1. Introduction

**Scheme 1:**
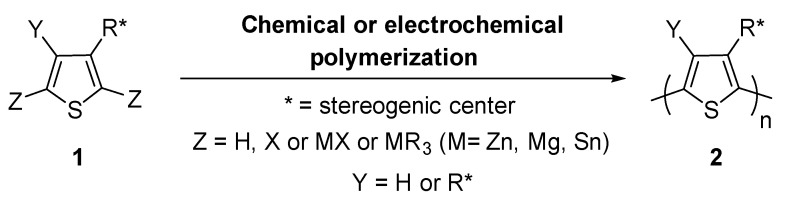
The general approach to conductive polymers **2** from the monomeric units **1**.

The phenomenon of chirality plays a key role not only in plant and animal life, but also in the biological activity of drugs, food additives and agrochemicals [1,2,3,4,5]. In consequence, chiral structures are used very often as active components in pharmaceutical [6,7,8,9], agrochemical [10,11] and food formulations [12]. The last two decades have witnessed a growing demand for such derivatives as essential component of new materials including nonfunctionalized and functionalized polythiophenes (PTs). Interest in this group of synthetic polymeric materials comes from the hope that they may mimic the behavior of natural polymers and find application, for example, as chiral sensors, chiral catalysts and chiral chromatographic supports. When considering chiral PTs, the most readily available group should be derivatives of general formula **2** (Scheme 1) containing at least one substituent with a stereogenic center at the three-position (or at the four-position or both positions). They can be prepared by the chemical or electrochemical oxidation of the corresponding monomers of general formula **1** (unsubstituted or suitably functionalized in the two and/or five positions).

The synthetic non-regioselective routes to 2,5-coupled, 3-substituted polythiophenes provides the polythiophenes with a lower degree of HT (head-to-tail) type coupling in the main polymeric chain. They contain more regiochemical defects, which results in a distortion of the conjugated chain and a lower effective conjugation. Among these methods are chemical oxidative coupling using the Sugimoto protocol [13] and dehalogenative polycondensation using Ni(COD)_2_ [14]. The growing demand for the highly conductive materials applicable in electronic devices (i.e., field effect transistors, organic light-emitting diodes, photovoltaic cells, or sensors) induces the rapid developments in the regiospecific routes to regioregular PTs since the regioregular structure affects the improvement of their physico-chemical properties and their self-organization. Most of the synthetic approaches are based on the transition metal-catalyzed coupling reactions [15,16,17]. The first synthesis of regioregular head-to-tail coupled poly(3-alkylthiophenes) (PATs) was reported by McCullough and Lowe [18]. In this approach, the starting material is 2-bromo-3-substituted thiophene, which is selectively metalated with lithium diisopropylamide (LDA) and then treated with magnesium bromide, which led to the formation of the corresponding Grignard reagents. Their coupling reaction is catalyzed by Ni(dppp)Cl_2_. The modification of this method, developed by McCollough, is the GRIM metathesis procedure, which requires the use of a 2,5-dibromo-substituted thiophene derivative. The substrate reacts with the Grignard reagent, forming the corresponding thienyl magnesium halides, which then undergo a Ni-catalyzed coupling reaction. The polymerization proceeds via a living chain growth polycondensation mechanism that was independently discovered by Yokozawa and McCullough in 2004 [19,20]. Another regiospecific polymerization route that provides head-to-tail (HT) coupling products is the so-called Rieke method. It involves Ni-catalyzed coupling of organozinc halides generated in situ from 2,5-dihalogented thiophene derivatives by reactive forms of zinc [21]. The exception is when the palladium catalyst [Pd(PPh_3_)_4_] is utilized in this reaction of organozinc species, leading to a regiorandom type of coupling. 

All the above-mentioned regiospecific polymerizations giving access to a wide range of 3-substituted polythiophenes with enhanced chiroptical properties require the use of a Ni-based catalyst and are based on the initial generation of organozinc or organomagnesium halides. 

In turn, the Pd-catalyzed HT-coupling based polymerizations were also developed. These pathways demonstrate the good efficiency in Suzuki or Stille coupling reactions using boro- or organotin compounds generated from the starting 2-monohalo-thiophene derivatives. The Pd-catalyzed Stille or Suzuki polymerizations have already been found to proceed via step-growth polymerization and by a non-controlled mechanism providing the polymers with the lower polydispersity [15,22]. It should be noted that the organotin or organoboronic derivatives should be prepared under cryogenic conditions and they should be isolated prior to the next step.

Advances in the development of the efficient polymerization techniques leading to well-ordered conjugated polymers are based on some modifications, mostly in the Kumada-based polycondensation, which uses the internal initiator/catalyst and as well the recently applied external initiator species [23,24]. Kumada catalyst transfer condensative polymerization, being a modified GRIM method, is a controlled chain-growth *polymerization* that requires the use of a Ni-based chain initiator/catalyst. 

The pool of controlled chain grow polymerizations complements a Pd-RuPhos protocol that is Pd-catalyzed Negishi coupling polymerization between organozinc compounds [25].

Dehydrohalogenative polycondensation is the other example of Ni-catalyzed [Ni(dppp)Cl_2_] polymerization, which requires the use of a mono-halogenated thiophene substrate and a Knochel–Hauser base to proceed at room temperature with high regioregular couplings [26,27,28].

The other strategy to synthesize regioregular polythiophenes, apart from the selection of a methodology distinguished by the type of metal catalyst, the reaction conditions and the control over the polymerization of monomeric units, relies on designing the symmetrical units. In this way, regiochemically defined polythiophenes could be obtained from the units that contain the spacer block between 3-substituted thiophenes. Similarly, HH-TT coupled PATs and TT-HH PATs are accessible from regiochemically defined bithiophenes, i.e., the HH-dimer of substituted thiophene or TT dimers and synthesized via one of the non-regiospecific methods ( i.e. FeCl_3_-mediated chemical polymerization). 

These studies were summarized less or more briefly, occasionally, as part of reviews on chiral conductive polymers [29,30,31,32,33]. It is obvious that in all the studies devoted to the synthetic and structural aspects in the chemistry of chiral PTs, the preparation of the appropriately designed monomeric substrate constitutes an indispensable opening stage. Reading the original chemical literature gives the impression that the topic is treated quite briefly and without taking into account sufficient experimental details, the knowledge of which should be more or less helpful for all researchers in this field, especially for beginners entering this fascinating and rapidly developing topic. Therefore, the main focus of this mini-review is to extensively discuss the applied synthetic approaches extracted from the original publications that have been published since the first article on this topic in 1988 [34]. Taking into accounts the analysis of the substrate requirements of the mentioned polymerization methods, we propose the organization of the manuscript based on the type of substitution in monomeric precursors. Below, one by one, we will present protocols leading to optically active (or in some cases to racemic) thiophene derivatives of general structure **1:**

(a) monothiophenes substituted at the three-position of the ring with a group containing a stereogenic center or stereogenic axis;
(b) monothiophenes substituted at the three-position of the ring with a group containing a stereogenic center and at once functionalized at the two and/or five positions;
(c) monothiophenes substituted at the three and four positions with a group(s) containing a stereogenic center and nonfunctionalized or functionalized at the two and/or five positions;
(d) β-(mono or di) substituted bithiophenes functionalized with a substituent containing a stereogenic carbon atom or a stereogenic heteroatom;
(e) other (oligo)thiophenes functionalized with a substituent containing a stereogenic carbon atom or a stereogenic heteroatom.

## 2. Syntheses of 3-Substituted Monothiophenes Functionalized with a Substituent Containing a Stereogenic Center or a Stereogenic Axis

### 2.1. Syntheses of 3-Substituted Monothiophenes Nonfunctionalized in the Two and Five Positions

Due to the growing interest in the design of conducting polythiophenes with improved electrical and optical properties, mechanical, chemical and thermal stability, the research devoted to the synthesis of novel, diversely substituted monomeric units, for example those with the chiral 2-methylbutyl group. These functions are introduced into the structures while designing chiral nematic liquid crystals. 

**Scheme 2:**
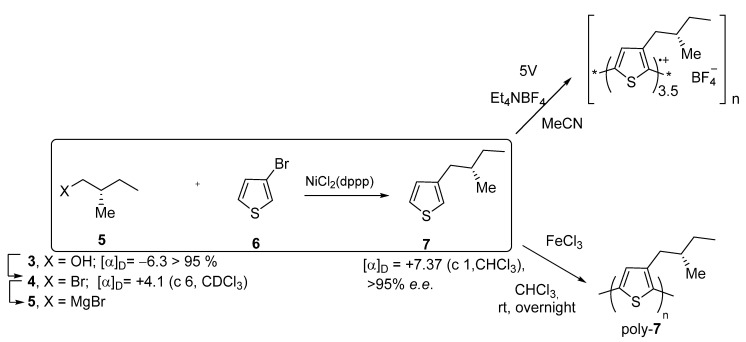
Synthesis of 3-[(*S*)-2-methylbutyl]thiophene **7** by the cross-coupling reaction of (*S*)-(2-methylbutyl)magnesium bromide **5** with 3-bromothiophene **6** and the subsequent electro- [35] and chemical polymerizations [37].

With a view to combining the liquid crystalline structure with a conductive polythiophene backbone, 3-(*S*)-2-methylbutylthiophene monomer **7** was synthesized [35] by reacting the generated from (*S*)-(+)-1-bromo-2-methylbutane **4** Grignard reagent **5** with 3 -bromothiophene **6** (Scheme 2) following the literature report devoted to a general method for the alkylation and arylation of haloheterocyclic compounds, which occurs in the presence of a catalytic quantity of [NiCl_2_(dppp)] (where dppp stands for Ph_2_ P(CH_2_)_3_PPh_2_) [36]. Later on, this procedure, carried out with commercially available 1-bromo-2-methylbutane (Aldrich) having [α]_D_ = +4.5, c 5, CHCl_3_, was found to give thiophene **7,** purified by distillation, with [α]_D_ = +7.37 (neat) in 58% yield [37]. There are also two other reports on the use of this protocol [38,39] but without giving any details of yield and specific rotation. 

The monomer **7** (0.25 M solution) was oxidatively electropolymerized with an applied potential of 5 V, in a two-electrode cell consisting of a glassy-carbon working electrode (0.26 cm^2^), a platinum wire counter electrode, in a dry MeCN and the Et_4_NBF_4_ (0.25 M) solution [35].

The chiral ordering in an aggregated conjugated poly{3-(*S*)-[2-methylbutyl]thiophene} was examined too after producing the polymeric materials using the ferric chloride oxidative method [37]. The doping effect prior to aggregation along with the doping level was explored to find the factors contributing to control chiral arrangement in conjugated polymer aggregates. 

Moreover, the studies on the polymerization also comprised their regioregular polymeric analogues. First, the synthesis of HT regioregular 2,5 coupled poly-3-(*S*)-2-methylbutylthiophene was reported by Langeveld-Voss et al. [40]. Then, the regioregular polymer was prepared with a Ni(dppp)Cl_2_ catalyst using the Grignard metathesis developed by McCullough ][38,41] However, this procedure requires the preparation of 2,5 di brominated precursor; therefore, this synthetic procedure will be mentioned below in the subsequent chapter. 

**Scheme 3:**
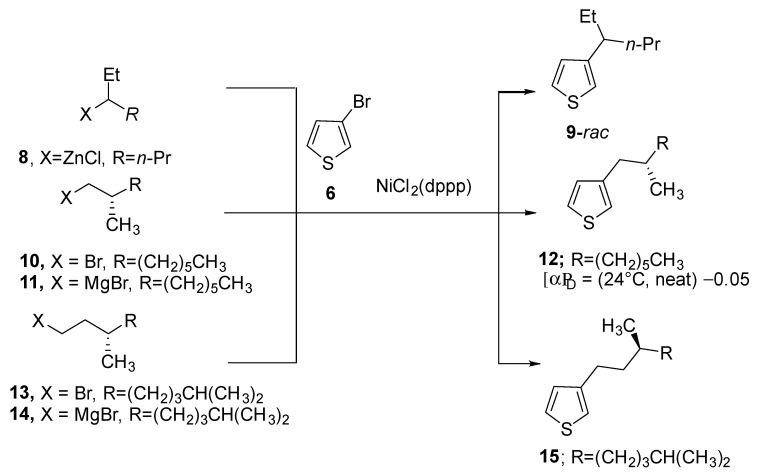
Synthesis of 3-[(1-ethyl)butyl]thiophene **9,** 3-[(*S*)-2-methyloctyl]thiophene **12** and 3-[(*S*)-3,7-dimethyloctyl]thiophene **15** by the cross-coupling reaction of 3-bromothiophene **6** with the corresponding alkylmagnesium or alkylzinc halides.

A recent China patent [42] lists among the product of cross-coupling of organozinc reagents and heterocyclic (pseudo)halides, racemic 3-(1-ethyl)butylthiophene **9** formed by the reaction of 3–bromothiophene **6** with hexan-3-ylzinc chloride **8** (Scheme 3).

Earlier, an EU patent owned by Nippon Telegraph and Telephone Corporation [43] reported the preparation 3-(*S*)-2-methyoctylthiophene monomer **12** achieved by coupling, generated from (*S*)-(+)-1-bromo-2-methyloctyl **10** Grignard reagent **11** with 3-bromothiophene **6** in the presence of a catalytic quantity of [NiCl_2_(dppp)] (Scheme 3). A similar approach was reported for the preparation of 3-(*S*)-3,7-dimethyoctylthiophene monomer **15** conducted by reacting the Grignard reagent **14** obtained from (*S*)-(+)-1-bromo-3,7-dimethyloctyl **13** with 3-bromothiophene **6**. Unfortunately, specific rotation data are not available (Scheme 3) [44]. 

The direct introduction of the substituent into the three position of the thiophene ring can be implemented by the Suzuki cross-coupling reaction, as illustrated in the synthesis of optically active (*R*)-3-(4-(4-ethyl-2-oxazolin-2-yl)phenyl)thiophene (EOPT) **18** [45]. 

**Scheme 4:**
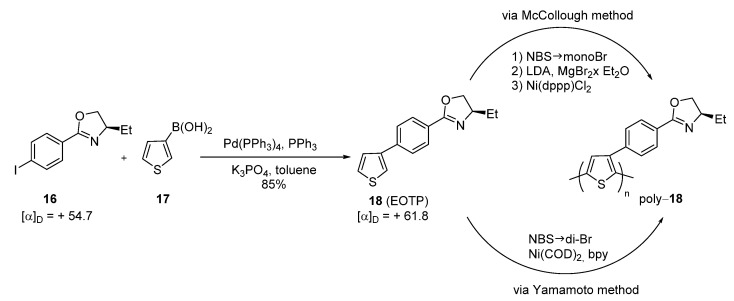
The cross-coupling of oxazoline **16** with 3-thiopheneboronic acid **17**. Syntheses of the regioregular and regiorandom poly-**18**.

The reaction occurred between 3-thiopheneboronic acid **17** and the oxazoline derivative **16** (prepared independently starting from ethyl 4-iodobenzoate and (*R*)-2-amino-1-butanol via an optically active hydroxy amide derivative [46]). The reaction carried out in the presence of K_3_PO_4_ in toluene using Pd(PPh_3_)_4_ as a catalyst gave **18** (EOPT) in 85% yield (Scheme 4) [47]. The enantiomeric excess of **18** was estimated to be greater than 99% using europium tris[3 -(trifluoromethylhydroxymethylene)-(+)-camphorate] (Eu(tfc)_3_) as a chiral shift reagent (^1^H NMR spectrum measured in CDCl_3_).

The regioregular polythiophene derived from monomeric thiophene **18** was synthesized according to a modified McCullough’s procedure when monobrominated thiophene derivative was used as a starting material. Dehalogenative polycondensation according to a Yamamoto protocol was used as well, successfully starting from the dibrominated analogue of the monomer **18**. It provided, however, the regiorandom polymer. The purpose of synthesizing the two polymers that differ in the regioregularity was to compare their chiroptical properties and to examine the influence of the regioregularity of the main chain on the induction of a chiral supramolecular aggregate in the chiral polythiophenes [47].

**Scheme 5:**
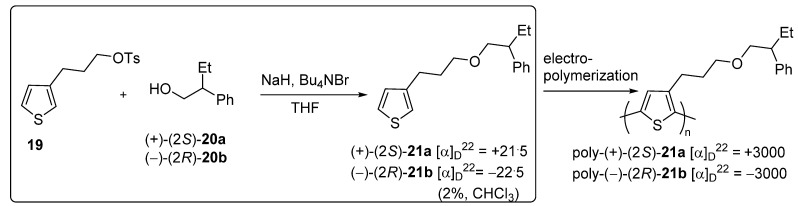
Synthesis of 3-[3-[(*S*)-2-phenylbutoxy)]propyl]thiophene **21a** and its enantiomer **21b**.

Among the optically active 3-alkylthiophene monomers, the most numerous groups are the compounds in which the alkyl group is functionalized at the terminal carbon atom by attaching a moiety containing a stereogenic center. The list of such compounds opens 3-[3-[(*S*)-2-phenylbutoxy)]propyl]thiophene **21a** and its enantiomer **21b** prepared by the reaction of the 3-(3′-thienyl)propyl toluene-*p*-sulfonate **19** with the enantiomers of 2-phenylbutanol **20a,b** (Scheme 5) [34,48]. The monomers were next electropolymerized, applying a current density of 2 mA cm^−2^ in a one-compartment three-electrode cell containing the monomer and NBu_4_PF_6_ in nitrobenzene. Chiral polythiophenes, poly-**21a** and poly-2**1b**, feature a high conductivity and were able to stereoselectively recognize the chiral anions used as doping agents during voltammetric cycles [34].

**Scheme 6:**
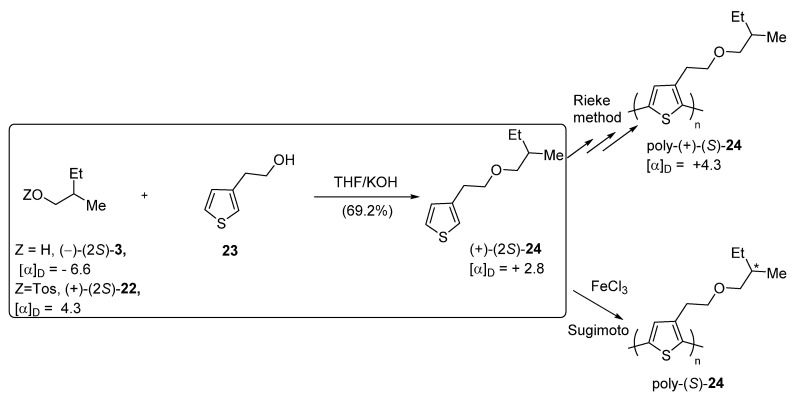
Synthesis of 3-[2-((*S*)-(1)-2-methylbutoxy)ethyl]thiophene **24** and its polymerizations.

The preparation of 3-[2-((*S*)-2-methylbutoxy)ethyl]thiophene **24** was, for the first time, mentioned as early as 1994 in a paper coming from the Meijer’s group [49]. They isolated it in 83% yield by reacting 2-(3-thienyl)ethanol **23** (obtained in 89% yield by a reduction of 3-thiopheneacetic acid with LiAIH_4_) with a tosylate **22** of the commercially available optically pure (*S*)-(−)-2-methyl-l-butanol **3**. This protocol was later referenced, without specifying characterization details, in three other publications by this team [40,50,51]. On the other hand, the paper by Ochiai et al. [52] contains the full experimental details of the protocol presented in Scheme 6. This protocol is given below. The monomer, after its conversion into the 2,5-dibromosubstituted derivative, was used to obtain an optically active regioregular 2,5-coupled analogue, poly(3-[2-((*S*)-2-methylbutoxy)ethyl]thiophene), containing a 93% HT head-to-tail linkage, according to the modified Rieke method [52]. The corresponding regiorandom poly-(*S*)-**24** was obtained by the FeCl_3_-oxidative polymerization of **24** [49].

**Scheme 7:**
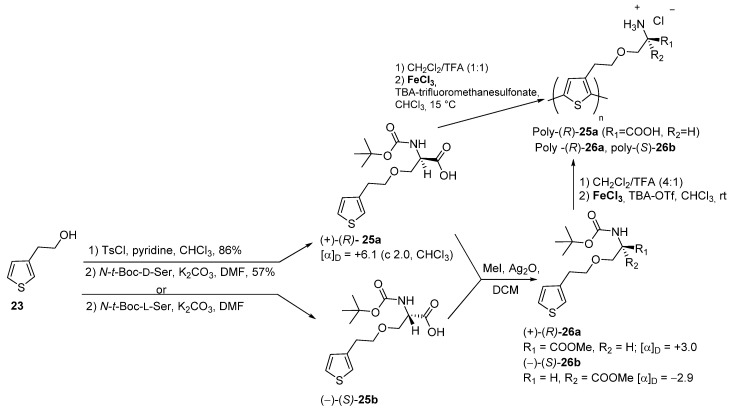
Synthesis of 2-*tert*-Butoxycarbonylamino-3-(2-thiophen-3-yl-ethoxy)-propionic acid (**25**), its conversion to methyl ester analogue (**26**) and the polymerizations using FeCl_3_-mediated oxidative method.

Another example of the condensation reaction aimed at appending a chiral moiety into the side chain of a 3-substituted thiophene derivative is the synthesis of the dextrorotatory enantiomer of thiophene containing an amino acid residue (+)-*R*- **25a**. The transformation begins with the conversion of 3-thienylethanol **23** to the corresponding tosylate and follows by the reaction with a protected unnatural amino acid, *N*-*t*-Boc-d-Ser (Scheme 7) [53]. A similar sequence of reactions using *N*-*t*-Boc-l-Ser gave the opposite enantiomer of ethoxythiophene (−)-*S*-**25b**. Their further transformations into the corresponding methyl esters **of** serine-substituted monothiophenes (+)-*R*-**26a**. or (−)-*S*-**26b** involve estrifications using Ag_2_O and MeI (Scheme 7) [54].

The *tert*-butoxycarbonyl group was removed (using a TFA/DCM solution) prior to polymerization. The free amino acid substituted thiophene monomer (*R*)-**25a** and its methyl ester analogue **26a** or its enantiomer **26b** were polymerized using a method reported by Sugimoto et al. using chemical oxidation with FeCl_3_ in chloroform. The water-soluble polymers, converted into the ammonium chloride salts, were precipitated by adding acetone [53,54].

**Scheme 8:**
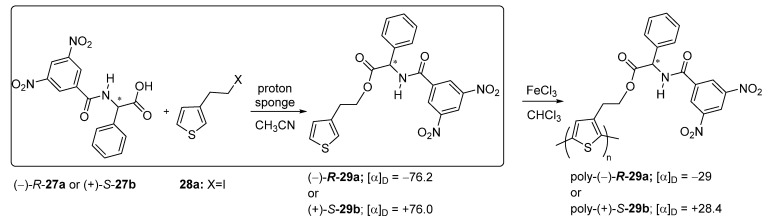
Synthesis of the monomers of thienyl phenylglycinate **29a** and **29b** and their polymerization.

Synthesis of the monomers (*R*)-(−)-2-(3-thienyl)ethyl *N*-(3′,5′-dinitrobenzoyl)- α-phenylglycinate **29a** and (*S*)-(+)-2-(3-thienyl)ethyl *N*-(3′,5′-dinitrobenzoyl)- α-phenylglycinate **29b** involves a similar approach based on the condensation of (*R*)-(−) or (*S*)-(+)-*N*-(3,5-dinitrobenzoyl)- α-phenylglycine **27a** or **27b** with 3-(2-iodoethyl)thiophene **28a** carried out in a basic medium (Scheme 8) [55]. Their enantiomeric excesses were supported by ^1^H NMR spectra measured in the presence of Eu(tfc)_3_. They are commonly used as chiral selectors in Pirkle’s stationary phases [56,57], applied in HPLC enantioselective analysis [58].

Once the monomers were isolated in good yields, namely, 51% for (*R*)-**29a** and 49% for (*S*)-**29b**, respectively, they were polymerized by oxidative coupling with FeCl_3_ in CHCl_3_ (Scheme 8). The optical activity of the polythiophenes was maintained as evidenced by the specific optical rotation values of [α]_D_^28^ = −29.0 (2.5, THF) for poly-(−)-(*R*)-**29a** and [α]_D_^28^ = +28.4 (2.5, THF) for poly-(+)-(*S*)-**29b**.

**Scheme 9:**
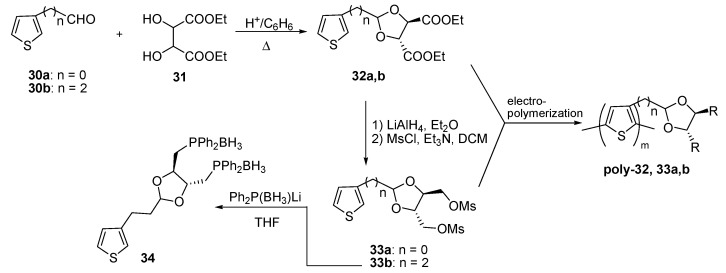
Synthesis of selected 3-substituted (poly)thiophenes with a chiral 1,3-dioxalane moiety.

Several 3-substituted thiophenes **(32a,b, 33a,b, 34b),** wherein thiophene rings are linked directly or via an alkyl spacer to a chiral acetal ring such as a 1,3-dioxalane having two stereogenic carbon atoms, were prepared using optically active diethyl tartrate **31** and 3-thiophenecarboxaldehyde **30a** or 3-thiophenepropanal **30b** as starting materials. Their synthesis is shown in Scheme 9 [59].

The compounds **32a,b** and **33a,b** were subjected to an anodic polymerization. It was reported that poly-(**33b**) was stable toward oxidative cycling. For poly-(**33**), the progressive electrochemical growth was observed in CH_2_Cl_2_ with 0.1M Bu_4_NBF_4_ as an inert electrolyte, while no polymerization occurred using CH_3_CN.

**Scheme 10:**
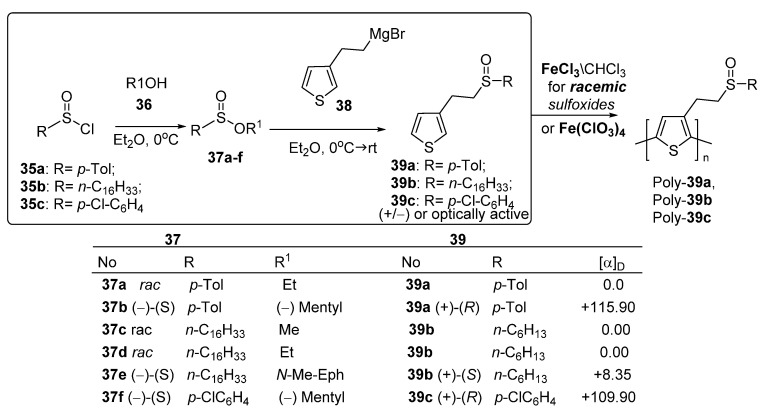
Synthesis of 2-(3′-thienyl)ethyl alkyl (aryl) sulfoxides **39a**–**c** by reacting sulfinates **37a**–**f** with 2-(3′-thienyl)ethylmagnesium bromide **38**.

**Scheme 11:**
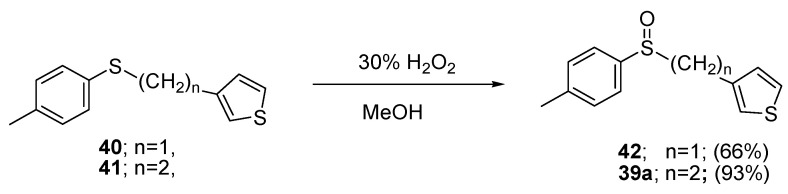
Oxidations of the 2- (3′-thienyl)alkyl *p*-tolyl sulfides **40** and **41** using a 30 wt% solution of hydrogen peroxide.

Recently, a few racemic and optically active 2-(3′-thienyl)ethyl alkyl (aryl) sulfoxides 3**9a**–**c** were prepared by reacting racemic or diasteromerically pure *O*-alkyl arene(alkyl) sulfinates **37a**–**f** (prepared in turn from arene(alkane)sulfinyl chlorides **35a**–**c** and an appropriate racemic or optically active alcohol **36**) with 2-(3′-thienyl)ethylmagnesium bromide **38** (Scheme 10). [60]. Moreover, sulfoxides **39a** and **42** were also obtained as racemates via the oxidation of the corresponding sulfides: 2- (3′-thienyl)ethyl *p*-tolyl **41** or 3-thienylmethyl *p*-tolyl **40** using a 30 wt% solution of hydrogen peroxide as an oxidizing reagent (Scheme 11) [60].

**Scheme 12:**
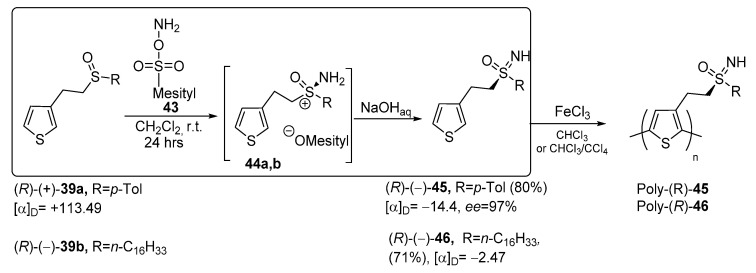
The preparation of 2- (3′-thienyl)ethyl *p*-tolyl(*n*-hexadecyl) sulfoximines by an imination with MSH and the subsequent polymerization.

The subsequent transformations led to the *N*-unprotected, optically active 2-(3′-thienyl)ethyl (*p*-toluene/*n*-hexadecyl) sulfoximines (*R*)-(−)**-45** and (*R*)-(−)-**46**. The imination of sulfoxides (*R*)-(+)-**39a** or (*R*)-(−)-**39c** using *O*-(mesitylsulfonyl)hydroxylamine (MSH) **43**, following the procedure developed by Tamura in 1972 [61] and Johnson in 1974 [62] (Scheme 12), proceeded in an enantioselective manner, and took place with the retention of the configuration at the stereogenic sulfur atom. It is interesting to note that this sulfoximine, having 97% *ee,* showed only a very weak, almost an imperceptible cotton effect in the circular dichroism spectrum recorded in methylene chloride. The racemic *n*-hexadecyl sulfoximine **46** was also obtained following this procedure.

**Scheme 13:**
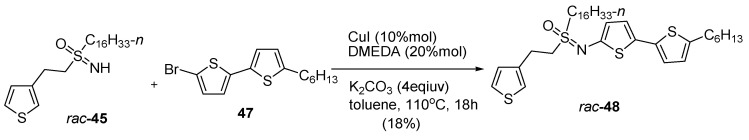
*N*-Bithienylation of racemic sulfoximine **45** catalyzed by Cu(I) DMEDA complex.

The *N* functionalization of racemic *n*-hexadecyl-2-(3′-thienyl)ethyl sulfoximine **45** was based on its reaction with 5-bromo-5′-hexyl-2,2′-bithiophene **47** in the presence of potassium carbonate and catalytic amounts of copper (I) iodide and *N,N*′-dimethylethane-1,2-diamine (DMEDA) (Scheme 13) [60]. This protocol, leading to *N*-bithiophene derivative **48** constitutes a modification of the procedure described by Bolm in 2005 [63], by which this coupling reaction was carried out with cesium carbonate as a base.

**Scheme 14:**
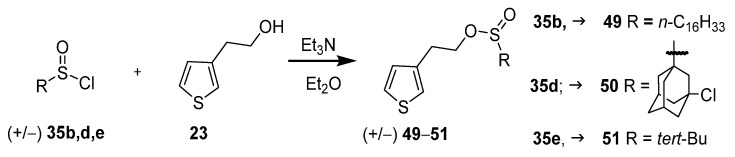
Condensation of alkanesulfinyl chlorides **35b** and **35d**,**e** with 2-(3′-thienyl)ethanol **20**.

The monothiophenes **49**–**51** containing a sulfonate function attached to the thiophene ring at the three-position through an ethylene linker were also prepared with the aim of their usage to obtain new chiral high molecular weight analogues. The condensation of the corresponding alkanesulfinyl chlorides **35b** and **35d**,**e** with 2-(3′-thienyl)ethanol **23** carried out in the presence of triethylamine in an ether solution gave the sulfonic esters in 50–87% yields (Scheme 14) [60].

Another class of monomers designed to synthesize new chiral polythiophenes in order to evaluate their properties are monothiophenes containing the substituent with a stereogenic phosphorus atom at the three-position of the ring. For this purpose, the phosphine oxide based monothiophene (−)-(*S*)-**53a** was synthesized first.

**Scheme 15:**
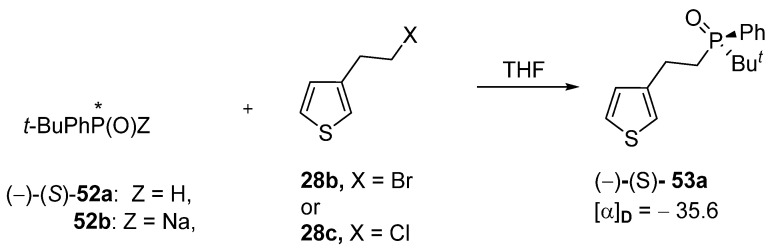
Alkylation of the metalated *tert*-butylphenylphosphine oxide **52b** with 2-(3′ thienyl)ethyl bromide **23b**.

Upon alkylation of the metalated *tert*-butylphenylphosphine oxide **52b** with 2-(3′ thienyl)ethyl bromide **28b** (or chloride **28c**), the optically active (*S*)-*tert*-butylphenyl-2-(3′-thiophene)ethylphosphine oxide **53a** could be isolated. The reaction proceeds with the retention of the configuration at a phosphinyl phosphorus atom and without detectable racemization (Scheme 15) [64]. This procedure was simultaneously patented [65,66] and applied for the preparation of racemic phosphine oxide *rac*-**53**.

**Scheme 16:**
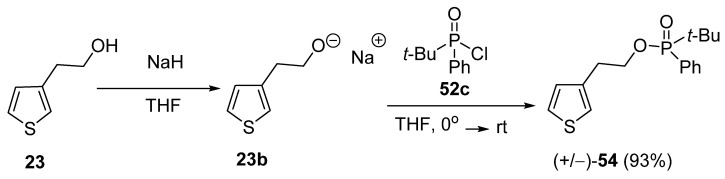
Reaction of *tert*-butylphenylphosphinyl chloride **52c** with sodium 2-(3′ thienyl)ethoxide **23b**.

Following the procedure of the reaction of sodium 2-(3′-thienyl)ethoxide **23b** with *tert*-butylphenylposhinyl chloride **52c**, the representative of the phosphonates, namely, racemic *O*-2-(3′-thienyl)ethyl *tert*-butylphenylphosphinate **54**, was prepared in 93% yield (Scheme 16) [64].

The above monomers, sulfoxides **39a**–**c**, sulfoximines (*R*)-(−)**-45** and (*R*)-(−)-**46,** alkanesulfonates **49**–**51,** phosphine oxides (−)-(*S*)-**53a** and phosphonates *rac***-54** were converted into the polythiophene analogues, in most cases by chemical oxidative polymerization according to a Sugimoto et al. protocol, or, in the case of the phosphorus based derivatives, via both the FeCl_3_-promoted method and electropolymerization [64]. Iron (III) chloride was the optimal oxidizing coupling agent, except for the polymerizations of optically active sulfoxides. Its use for polymerizing the enantiomerically pure sulfoxides failed in terms of isolating the optically active polymeric products. This was due to the release of gaseous HCl during polymerization, which led to racemization. The optical properties of the polythiophenes with different types of substitution were examined using UV–VIS and fluorescent spectroscopy, and the circular dichroism and polarimetric measurements were conducted to examine their chirality. The fluorescence quantum efficiency in the solutions of the regioirregularly 2,5-coupled polythiophenes reaches 24%.

The synthesis of the 3-substituted monothiophenes containing an alkyl side chain attached to the thiophene ring through a heteroatom was developed, as the presence of the heteroatom directly attached to the heterocyclic ring affects the properties of the monomers as well the products of their polymerizations.

**Scheme 17:**
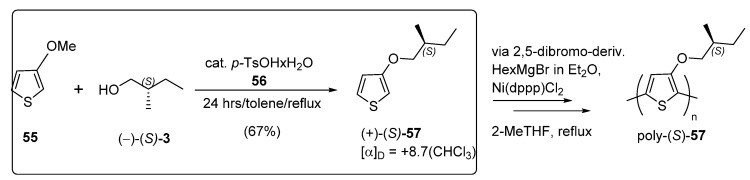
Acid–catalyzed reaction of 3-methoxythiophene **55** with (*S*)-(−)-2-methyl-1-butanol **3**.

One of the representatives is alkoxy substituted thiophene with an electro-donating substituent, optically active (+)-3-[(*S*)-(2-methylbutoxy)]thiophene (TOR*) **57,** which was prepared in 67% yield by the acid catalyzed reaction of 3-methoxythiophene **55** with (*S*)-(−)-2-methyl-1-butanol **3** refluxed in a toluene solution for 24 h in the presence of a catalytic amounts of *p*-toluenesulfonic acid monohydrate (Scheme 17) [67].

The monomer (*S*)-**57** was used to prepare novel, optically active oligothiophenes using synthetic methods suitable for obtaining regioregular head-to-tail and head-to-head/tail-to-tail derivatives (if starting from the bithiophene monomers). The chiral (*S*)-(2-methyl)butyl moiety was introduced into the three-position of the thiophene ring through heteroatoms, such as O as well S (present in the compound **58**) to prepare oligomeric regioregular HT-coupled oligomers being suitable models for the evaluation of the effect of the heteroatom presence in the side chain on the macromolecular aggregation and, consequently, on the chiroptical properties of the material in the solid state. The GRIM (Grignard metathesis) procedure, via a magnesium/bromine exchange, was adopted for the polymerization under a high regio control.

**Scheme 18:**
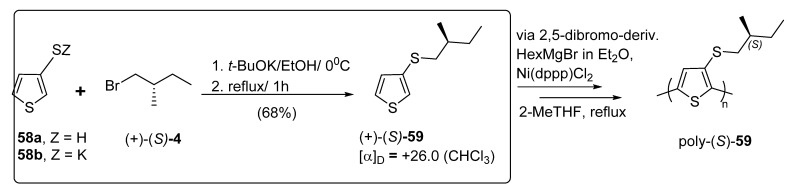
Alkylation of potassium 3-mercaptothiophenate **58b** by 1-bromo-2-methylbutane (*S*)-(+)-**4**.

In turn, the sulfur analogue of **57**, optically active (+)- 3-[(*S*)-2-methylbutylsulfanyl]thiophene **59,** was obtained by the alkylation of potassium 3-mercaptothiophenate **58b**, prepared in situ by treating the parent 3-thiophenethiol **58a** with potassium *tert*-butylate in ethanol, with the 1-bromo-2-methylbutane enantiomer, (+)-(*S*)-**4** (Scheme 18) [68]. The monomer was, similar to its oxygen analogue, polymerized using the GRIM method.

**Scheme 19:**
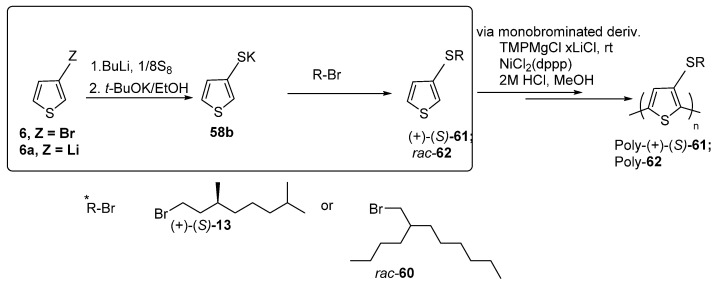
Alkylation of potassium 3-mercaptothiophenate **58b** by chiral bromo-alkanes **(13)** or **(60)** in the preparation of alkylsulfanylthiophenes and the route to their polymerization.

A similar approach was used for the preparation of optically active (+)-3- [(*S*)-3,7-dimethyloctylsulfanyl]thiophene **61** and a racemic 3-[2-butyloctyl] thioeter **62**. This synthetic route, reported originally by Barbarella and co-workers [69] begins with a lithium−halogen exchange reaction of 3-bromothiophene **6** followed by quenching with sulfur to give 3-thiophenethiol **58a,** which, after its conversion into the corresponding potassium thiolate **58b,** reacted with bromoalkanes [(+)-(*S*)-**13** or **60**] to provide facile access to the target 3-(alkylthio)thiophenes **61**,**62** (Scheme 19) **[70]**.

In the case of sulfides **61** and **62**, the dehydrohalogenative polycondensation method was used for their polymerization The monobrominated products were the starting materials for a Kumada polymerization procedure that produces highly regioregular HT polythiophenes in high yields In this procedure Knochel’s base [28,71] was used to deprotonate the monomer, and 0.5 mol % NiCl_2_(dppe) was applied as the catalyst. Poly-**61** and poly-**62** exhibited electrochemical behavior with reversible p-doping and dedoping processes. The helicity of chiral polymers was examined using the CD measurements taken in the solution and in the solid states. The CD measurements in solution showed strong bisignate cotton effects for the π–π* transitions in poly-(***S***)-**-61**, whereas no cotton effect was observed for poly-**62**. The addition of MeOH, a poor solvent, to the polymers resulted in an increased CD signal corresponding to the formation of an ordered helical aggregation.

### 2.2. Syntheses of 3-Substituted Monothiophenes Functionalized in the Two Position

**Scheme 20:**
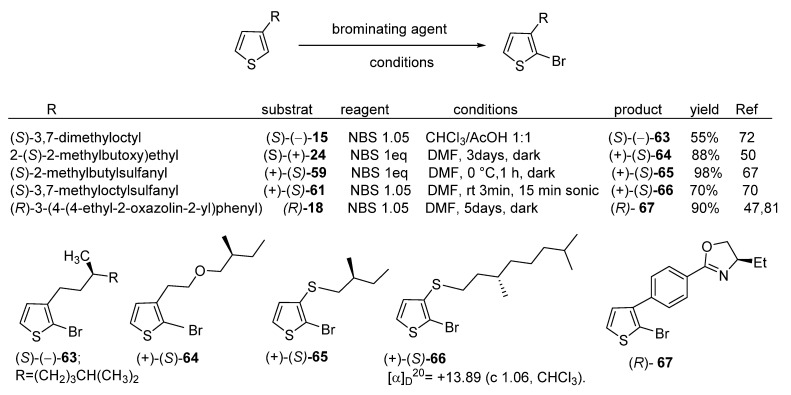
Selective brominations of thiophenes functionalized with a chiral alkyl group.

In this sub-class of monothiophenes substituted in the three-position with a substituent containing a stereogenic carbon atom or a stereogenic heteroatom and additionally functionalized in the two -position, the most abundant group are those with halogen substituents in the two- -position. Additionally, in turn, among them, 2-bromo derivatives were the most often obtained. They are typically prepared by the bromination of the parent 3-substituted monothiophenes mentioned above with *N*-bromosuccinimide (NBS) under selected conditions, as summarized in Scheme 20.

As an example, 3-[(−) 3-(*S*)-3,7-dimethyoctyl]thiophene **15** was converted to (−) 2–bromo-3-(*S*)-(3,7-dimethyoctyl)thiophene **63** [72,73,74]; (+)-3-[2-((*S*)-2- methylbutoxy)ethyl]thiophene **24** to (+)-2-bromo-3-[2-((*S*)-2- methylbutoxy)ethyl]thiophene **64[50]** [50,51,75]; (+)- 3-[(*S*) -2-methylbutylsulfanyl]thiophene **59** to the corresponding 2-bromo derivative **65** [67,68,70]; (+)-3- [(*S*)-3,7-dimethyloctylsulfanyl]thiophene **61** to 2-bromo derivative **66** [70] and (*R*)-3-(4-(4-ethyl-2-oxazolin-2-yl)phenyl)thiophene (EOPT) **18** to EOPT-Br **67** (Scheme 20) [47].

**Scheme 21:**
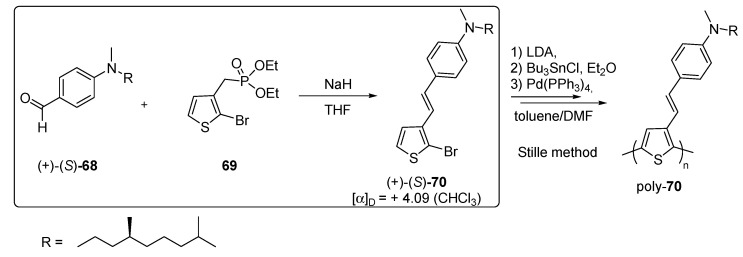
A Wittig–Horner reaction of the aldehyde **68** with 2-bromothiophenephosphonate **69**.

An interesting synthesis of (*S*)-(1)-2-bromo-3-[2-[4-[*N-*methyl-*N*-(3,7-dimethyloctyl)amino]phenyl]ethenyl]thiophene **70** was achieved by the coupling of the aldehyde **68** with 2-bromothiophenephosphonate **69** under a Wittig–Horner reaction (Scheme 21). ^1^H NMR spectroscopy confirmed that *trans*-**70** is formed exclusively [76]. The regioregular polythiophene poly-**70** was prepared by the polycondensation of **70** via a Stille coupling with Pd(PPh_3_)_4_ as a catalyst. Their physicochemical measurements indicated that the sidechain influences on the spectroscopic (absorption, emission) and electrochemical behavior and that the sidechain chirally stacks in conditions in which the polymer backbone aggregates. This indicates the ability of inducing a (chiral) lamellar organization of conjugated moieties, present in their sidechain.

**Scheme 22:**
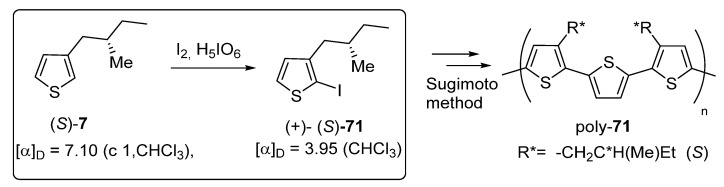
Iodination of 3-[(*S*)-(+)-2-methylbutyl]thiophene **7**.

The selective iodination of optically active thiophenes **7** and **24** was elaborated while synthesizing (*S*)-(+)-2-iodo-3-(2-methylbutyl)thiophene **71** [39] and (*S*)-2-iodo- 3-[2-methylbutoxy)ethyl]thiophene **72** [49]. The iodination of the optically active 3-[(*S*)-(+)-2-methylbutyl]thiophene **7** followed the method of Suzuki [77] (Scheme 22). The monoiodo-substituted thiophene (*S*)-**71** was used to prepare the terthiophene derivative, which served as a monomeric unit in the formation of the copolymer poly**-71** via the FeCl_3_-mediated oxidative coupling.

**Scheme 23:**
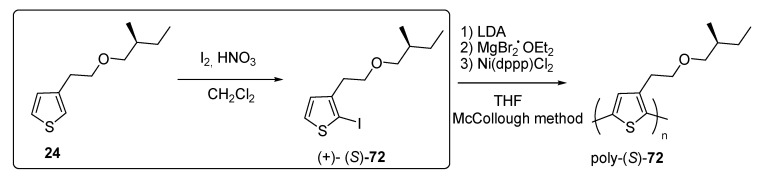
Iodination of 2-((*S*)-2-methylbutoxy)ethyl]thiophene **24**.

2-((*S*)-2-Methylbutoxy)ethyl]thiophene **24** was iodinated at the two-position using I_2_, in HNO_3_, to give (*S*)- 2-iodo- 3-[2-methylbutoxy)ethyl]thiophene **72** in 47% yield after distillation (Scheme 23). The latter was transformed into the polymeric analogue poly-(*S*)-**72** using McCullough’s method. The corresponding regiorandom isomer of poly**-72** was also obtained by the FeCl_3_-mediated oxidative polymerization of **24** at −20 °C.

### 2.3. Syntheses of 3-Substituted Monothiophenes Functionalized in the Two and Five Positions

**Scheme 24:**
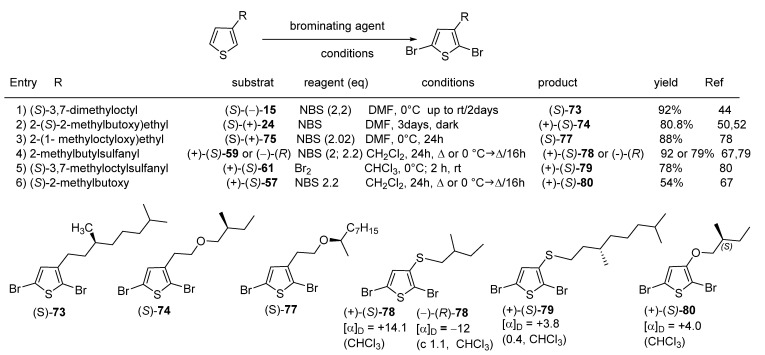
Double bromination of optically active thiophene derivatives with NBS or Br_2_.

In this sub-class of monothiophenes substituted in the three-position with a substituent containing a stereogenic carbon atom or a stereogenic heteroatom, the halogen-functionalized compounds in the two- and five-positions of the thiophene ring constitute the most abundant group. Additionally, in turn, among them, dibromo derivatives were most often prepared. They are prepared most easily by the dibromination of the parent 3-substituted using *N*-bromosuccinimide (NBS) or bromine. In this manner, 3-[(−) 3-(*S*)-3,7-dimethyoctyl]thiophene **15** was converted to -(−) 2,5 –dibromo-3-(*S*)-(3,7-dimethyoctyl)thiophene **73** (Scheme 24, Entry 1) [44]. Unfortunately, specific rotation data are not available.

Similarly, (+)-3-[2-((*S*)-2- methylbutoxy)ethyl]thiophene **24** was converted to (+)-2,5-dibromo-3-[2-((*S*)-2- methylbutoxy)ethyl]thiophene **74** (Scheme 24, Entry 2) [50,52].

An analogous reaction of optically active 3-[2-(1- methyloctyloxy)ethyl]thiophene **75** afforded either the monobromothiophene **75** or dibromothiophene **77,** dependent on the amount of NBS used (Scheme 24, Entry 3) [78]. Fully regioselective dibromination was again observed for 3-[2-methylbutylsulfanyl]thiophene **59**, giving the corresponding 2.5-dibromo derivative **78** (Scheme 24, Entry 4) [67,68,70,79]; (+)-3- [(*S*)-3,7-dimethyloctylsulfanyl]thiophene **61,** leading to 2,5-dibromo derivative **79** (Scheme 24, Entry 5) [80] and (+)-3-[(*S*) -2-methylbutoxyl]thiophene **57,** giving the corresponding 2.5-dibromo derivative **80** (Scheme 24, Entry 6) [67].

**Scheme 25:**
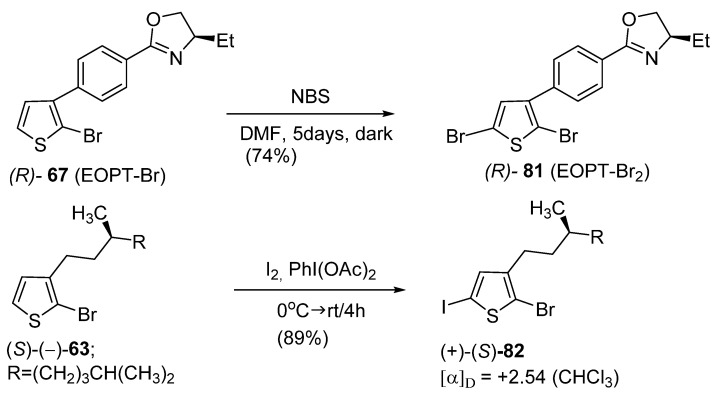
Bromination of optically active bromothiophene **67** with NBS and iodination of bromothiophene **63**.

The sequential halogenation of the 3-substituted monothiophenes was also conducted. Thus, 2,5-dibromo-(*R*)-3-(4-(4-ethyl-2-oxazolin-2-yl)phenyl)thiophene (EOPT-Br_2_) **81** was obtained by the subsequent bromination of **(***R***)-67** (EOPT-Br) at the five-position of the thiophene ring (Scheme 25**)** under similar conditions [47,61,81]. In turn, (+)-2-bromo-5-iodo-3-((*S*)-3,7- dimethyloctyl)thiophene (*S*)-**82** was prepared by the iodination of 2-bromo-3-((*S*)-3,7-dimethyloctyl)thiophene (*S*)-**63** with iodine in the presence of iodobenzene diacetate. (Scheme 25) [22,73,82,83,84].

**Scheme 26:**
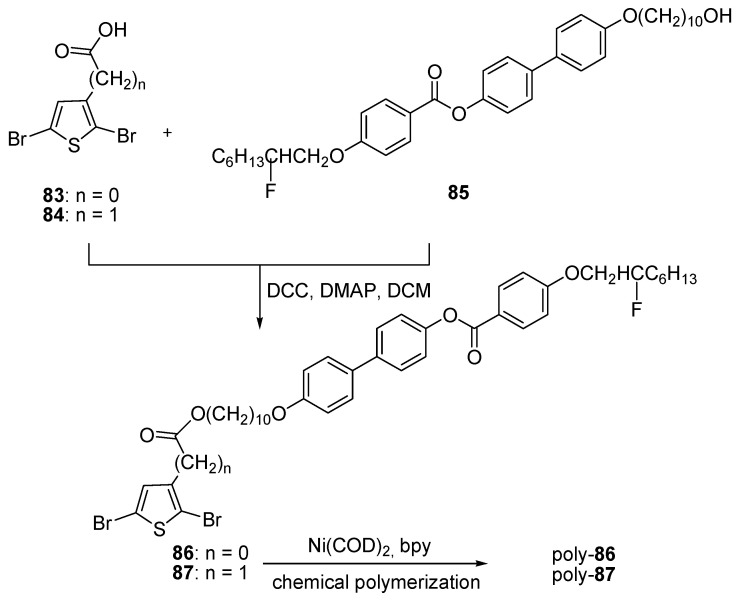
Coupling reaction of thiophenecarboxylic acids **83** and **84** with chiral alcohol **85**.

The other synthetic modifications concern the functionalization of the terminal reactive groups attached to the side chains in the three positions of the thiophene ring. This strategy is exemplified by the reaction of 2,3,5-trisubstituted monothiophenes having a functional group in the three-position permitting a coupling reaction with a chiral moiety, as illustrated in Scheme 26. The coupling reaction of 2,5-dibromothiophene-3-carboxylic acid **83** or 2,5-dibromothiophene-3-acetic acid **84** with 10-(4-(4′-(4′′-(2-fluorooctyloxy)phenylcarbonyloxy))-biphenoxy)decylol **85** occurred in the presence of dicyclohexylcarbodiimide (DCC) and 4-dimethyloaminopyridine (DMAP). The debrominative polycondensation of the resulting monomers **86**, **87** using the Ni zerovalent catalyst and the bis-chelating bipyridine ligand was applied for the preparation of the polymers, containing liquid crystalline side chains, poly-**86** and poly-**87** [85].

**Scheme 27:**
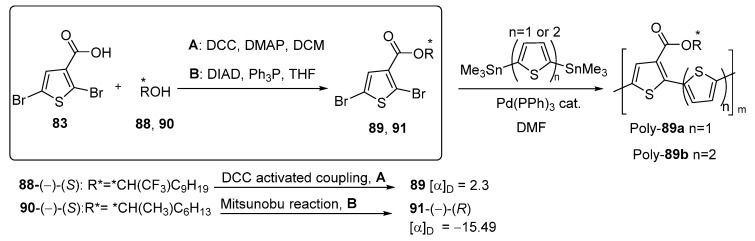
Esterification of 2,5-dibromothiophene-3-carboxylic acid **83** with optically active alcohols **88 and 90**. Copolymerization reaction via Stille coupling method.

A similar esterification reaction of 2,5-dibromothiophene-3-carboxylic acid **83** and (*S*)-1,1,1-trifluoro-undecan-2-ol **98** was found to afford the chiral ester **89**, while the Mitsunobu reaction of 2,5-dibromothiophene-3- carboxylic acid **83** and *(S*)- 2-octanol **90** gave the corresponding ester **91,** as shown in Scheme 27 [86]. The stereogenic center in (*S*)-2-octanol **90** was converted into (*R*)-configuration in the ester **91**, having [α]_D_ = −15.49, without racemization, since this reaction occurs with the SN_2_-type Walden inversion (Scheme 27) [87].

The monomer **89** was used for copolymerization with 2,5-bistannylated thiophene or 2′,5′’-distanyllated bithiophene via a Pd-catalyzed Stille polycondensation reaction. The chiral aggregation for the obtained polymers, poly-**89a** and poly-**89b,** was investigated using CD spectroscopy measurements.

**Scheme 28:**
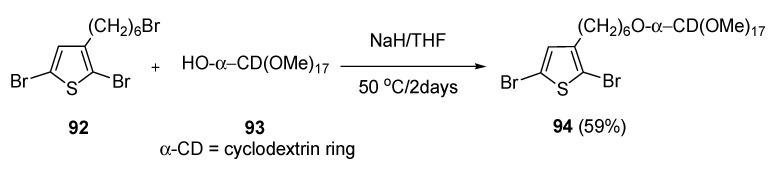
Synthesis of the permethyl-α-cyclodextrin **94** modified with a 2,5 dibromothiophene ring.

2,5-Dibromo-3-(6-bromohexyl)thiophene **92** reacted with permetylated 6-*O*-monohydroxy-α-cyclodextrin **93** in the presence of sodium hydride to give the bulky permethyl-α-cyclodextrin **94** in which the thiophene ring is attached through a hexamethylene linker (Scheme 28) [88].

## 3. Syntheses of 3,4-Disubstituted Monothiophenes

**Scheme 29:**
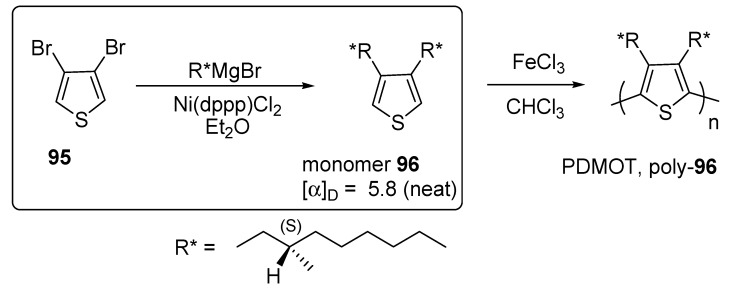
The approach to (poly)3,4-bis[(*S*)-2-methyloctyl]thiophene.

The first dialkyl-substituted chiral polythiophene, PDMOT: poly-{3,4-bis[(*S*)-2-methyloctyl]thiophene}, poly-**96** was synthesized in 2002 [89]. The synthesis of its precursor, the monomer **96**: 3,4-bis[(*S*)-2-methyloctyl]thiophene, was thereby developed and reported therein. It comprised the Kumada type coupling of 3,4-dibromothiophene **95** with (*S*)-2-methyloctylmagnesium bromide catalyzed by a Ni(II) catalyst to provide the product in 21% yield (Scheme 29). The precursor **96** was polymerized according to the Sugimoto protocol via the oxidative method. Interestingly, the UV–vis spectrum of PDMOT showed the absorption band at a shorter wavelength (λmax), 318 nm in chloroform, compared to monoalkyl-substituted polythiophenes. It was suggested that it could be due to the electronic effects of the electron donating alkyl side chains or due to their steric demands. It was assumed that the steric effects of the two branched chiral alkyl side chains in the polythiophene are responsible for an unusual CD spectrum and the significant thermo- and solvato-driven effects observed in the CD spectra.

While exploring the EDOT (ethylenedioxythiophene) derivatives, it was shown that the Mitsunobu reaction was an efficient route for the synthesis of mono- and disubstituted EDOTs and 3,4-propylenedioxythiophenes (ProDOTs) [90,91]. However, the one drawback it suffered from was a moderate yield, which prevailed in the case of the Mitsunobu reaction for synthesizing disubstituted EDOTs.

Later, the same research group demonstrated the high yielding alternative for the synthesis of the EDOT monomers achieved by transetherification of 3,4-dimethoxythiophene **103** with (chiral) glycols **104** [92]. The chiral 3,4-ethylenedioxythiophenes EDOTs, having two substituents at the ethylene bridge, containing two stereogenic centers in their units, were designed to ensure high regioregularity in the subsequent polymerization to produce chiral PEDOTs. In fact, it was also demonstrated that the stereochemistry of the monomers affected the electronic properties of the corresponding chiral PEDOT derivatives.

**Scheme 30:**
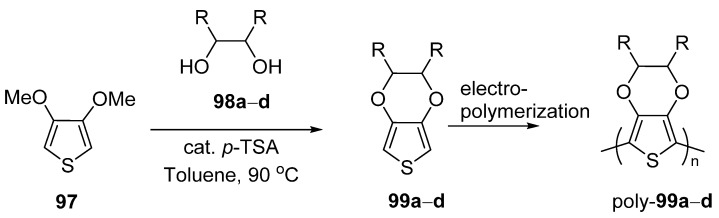
Novel EDOTs **99b**–**d** synthesis via the acid-catalyzed transetherification reaction and their use to obtain the corresponding PEDOTs.

The general synthetic protocol comprises the reactions of 3,4-dimethoxythiophene **97** with glycol derivatives **98a**–**d** catalyzed by *p*-toluenesulfonic acid (Scheme 30).

The reaction yield was lowered (20%) when (*meso*)-2,3-butanediol **98a** was used in preparing EDOT **99a**, (entry 1, Table 1). The stereochemistry of the starting 1,2-diols did not affect the yields in forming the resulting EDOTs in which the configurations are fully retained (entries 2–4: 73–82%; entries 5–9: 52–68%). All novel disubstituted EDOT derivatives **98a**,**d** were polymerized to PEDOTs using potentiodynamic electrooxidation.

A similar method, based on the transetherification of diols with 3,4-dimethoxythiophene **97**, was also applied for the synthesis of monomers **102a**,**b**, enantiomerically pure disubstituted 3,4-propylenedioxythiophenes containing (2*S*)-methylbutyl and (2*S*)-ethylhexyl sidechains [93].

**Scheme 31:**
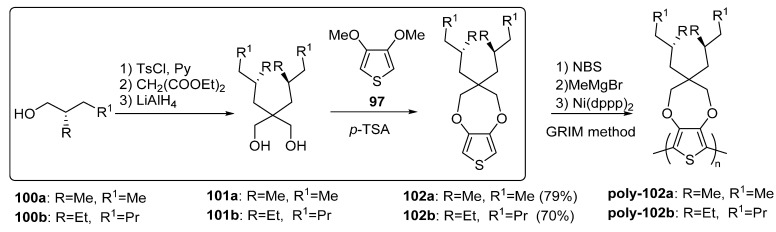
Synthesis of chiral 3,4-propylenedioxythiophenes ProDOTs **102a,b** from enantiomerically pure (2*S*)-methylbutanol **100a** and (2*S*)-ethylhexanol **100b**.

The procedure used for the synthesis of monomeric 3,4-propylenedioxythiophenes was modified in respect to a protocol for the racemic analogues described earlier [94]. The chiral alcohols, (2*S*)-methylbutanol **100a** and (2*S*)-ethylhexanol **100b**, were converted into the corresponding tosylates. The resulting products reacted with diethyl malonate anion to give malonic acid esters. Upon reduction with LiAlH_4_, the corresponding diols **101a**,**b** were obtained. Transetherification of the latter with 3,4-dimethoxythiophene **97** afforded oxidatively polymerizable ProDOT-((2*S*)-methylbutyl)_2_ (**102a**) and ProDOT-((2*S*)-ethylhexyl)_2_ (**102b**) (Scheme 31). To obtain the corresponding polymers with enantiomerically pure sidechains, bromination with NBS preceded a nickel-catalyzed Grignard metathesis polymerization. The optical properties of the polymer poly-**102b** were studied using UV–vis absorption, fluorescence and CD spectroscopy, and compared to the chiral methylbutyl derivative poly-**102a**. The aggregation process was followed by CD spectroscopy, which indicated that the two polymer forms chiral helical aggregates were similar to other chiral alkyl polythiophenes. Notably, the polymer poly-**102b** was recognized to have a fluorescence with a high quantum yield of 0.43 in xylene solution, while in the xylene/DMFmixture, along with a small decrease in the fluorescence quantum yield down to 0.28, a red-shift of the absorbance in the UV–vis spectrum was observed. Upon the addition of the poor solvent, a red-shift of the absorption spectrum and the vibronic features suggested more ordered, planar chains.

Among the class of π-conjugated polymers, polythiophenes with two alkylsulfanyl substituents in the β positions were also subject to detailed research. This class of polymers are expected to act as sensors for palladium or ruthenium by the quenching of their fluorescence to a greater extent than the quenching observed for nonthio functionalized polythiophenes.

**Scheme 32:**
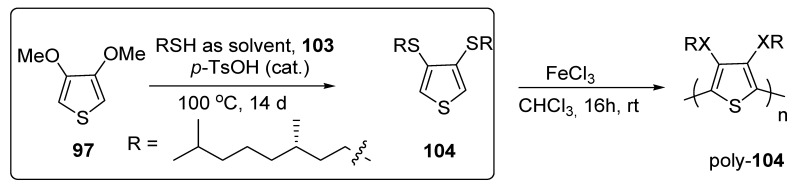
Synthesis of monomer 3,4-bis(3,7- dimethyloctylthio)thiophene **104** and its high molecular weight analogue poly-**104**.

The representative for the studies was 3,4-bis(3,7- dimethyloctylthio)thiophene, **104,** with a structure designed to possess well solubilizing chains attached to sulfur atoms, which was first synthesized and was then polymerized by oxidative coupling with iron(III) chloride, to give polythiophene derivative poly-**104** (Scheme 32) [95].

In the further studies, chiral bis-alkyloxo-substituted **111** and bis-alkylthio polythienylethynylene **112** derivatives have been synthesized to examine their affinity to coordinate transition metals and the influence of aggregation on the quenching of fluorescence upon coordination to metals [95]. The synthetic pathway started with the iodination of **105** and **106** at the two- and five-position by *N*-iodosuccinimide (NIS). The iodinated products, **107** and **108**, were acetylated by a reaction with trimethylsilylacetylene catalyzed by tetrakis(triphenylphosphine)palladium(0) and copper(I) iodide. The TMS-protected intermediate was treated with tetrabutylammonium fluoride to remove the silyl group to give **109** and **110**.

**Scheme 33:**
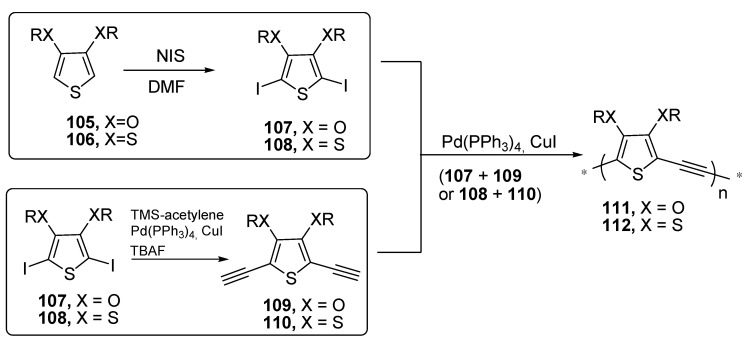
Synthesis of monomers: 3,4-bis(3(*S*),7-dimethyloctyloxy)-2,5-diiodothiophene **107**, 3,4-bis(3(*S*),7-dimethyloctylthio)-2,5-diiodothiophene **108**, 3,4-bis(3(*S*),7-dimethyloctyloxy)-2,5-diethynylthiophene **109**, 3,4-bis(3(*S*),7-dimethyloctylthio)-2,5-diethynylthiophene **110** and the products of their copolymerization **111** and **112**.

These monomers, **109** and **110,** and iodinated monothiophene monomers **107** and **108** were subjected to Pd-catalyzed polycondensation to give the corresponding polymers **111** and **112** (Scheme 33).

## 4. Syntheses of β-Substituted Bithiophenes Functionalized with a Substituent Containing a Stereogenic Carbon Atom or a Stereogenic Heteroatom

**Figure 1:**
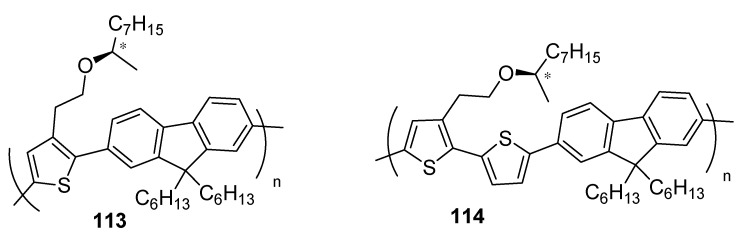
Structures of copolymers with the chiral side chains.

**Scheme 34:**
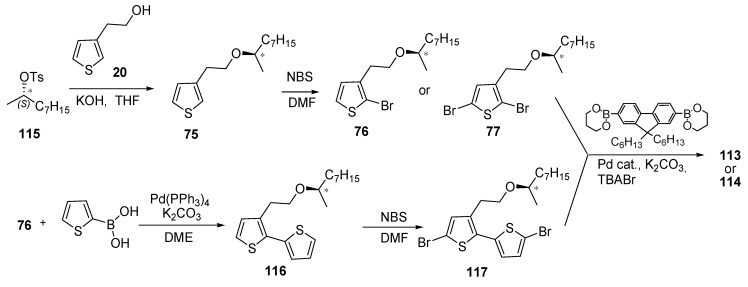
Synthesis of 2,5-dibromo-3-{2-[(*S*)-(+)-1-methyloctyloxy]ethyl}-2,2′-bithiophene (**117**) as a precursor for copolymerization with 9,9-dihexylfluorene-2,7-bis(trimethylene boronate).

The polyfluorene–thiophene copolymers have attracted much attention due to their optical and electrical properties. One of the components in the π-conjugated co-polymer, a polyfluorene derivative, is responsible for high photoluminescence quantum yields, good solubility and an interesting thermotropic liquid crystalline nature. The second component, polythiophene derivative, provides good processability and unique properties that are tunable by various side chains. The polyfluorene–thiophene copolymers, such as: poly[9,9-dihexylfluorenealt-(3-{2-[(*S*)-(+)-1-methyloctyloxy]ethyl*}*thiophene)] (**113**) and poly[9,9-dihexylfluorene-alt-(3-*{*2-[(*S*)-(+)-1-methyloctyloxy]ethyl*}*-2,2′-bithiophene)] (**114**) (Figure 1) [78], having chiral side chains located, in each of the model compound, in a different distance from the fluorene units, were obtained and screened for their optical properties. The synthesis of **113** and **114** was achieved via the Pd catalyzed Suzuki coupling method by reacting dibromides **77** and **124**, respectively, with 9,9-dihexylfluorene-2,7-bis(trimethylene boronate) (Scheme 34). Both copolymers exhibited a solvatochromism that was related to the strong self-organization of the copolymers. In the circular dichroism spectra, strong bisignate Cotton effects were observed in the methanol/chloroform mixed solutions.

The monomers for **113** and **114**, 2,5-dibromo--3-{2-[(*S*)-(+)-1-methyloctyloxy]ethyl}thiophene **77** and 2,5-dibromo-3-{2-[(*S*)-(+)-1-methyloctyloxy]ethyl}2,2′-bithiophene **117,** respectively, were synthesized as shown in Scheme 34 [78,96].

The β,β’-Disubstituted bithiophene with two (*S*)-2-methylbutylsulfanyl substituents was prepared and applied as a monomer for chemical (FeCl_3_) or electrochemical polymerization, leading to a regioregular head-to-head/tail-to-tail poly(β,β’-disubstituted bithiophene) [97].

**Scheme 35:**
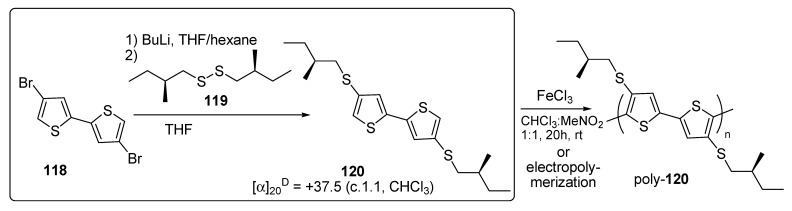
Synthesis of monomer **120** and its conversion to poly[4,4′-bis[(*S*)-2-methylbutylsulfanyl]-2,2′-bithiophene poly-**120**.

The synthesis of (+)-4,4′-bis[(*S*)-2-methylbutylsulfanyl]-2,2′-bithiophene **120** (Scheme 35) comprised the bromine–lithium exchange when 4,4′-dibromo-2,2′-bithiophene **118** [98] was treated with butyl lithium, followed by a reaction with (+)-bis[(*S*)-2-methylbutyl]disulfide **119**.

The synthesis of the modified bithiophenes, bearing one-alkylsulfanyl substituent in the β-position and one other substituent in the β’-position, was further developed [68]. The purpose of these studies was to gain a better insight into the reactivity of *β*-alkylsulfanyl-substituted bithiophenes as they were converted into the octithiophene derivatives via one-pot oxidative coupling with FeCl_3_.

**Scheme 36:**
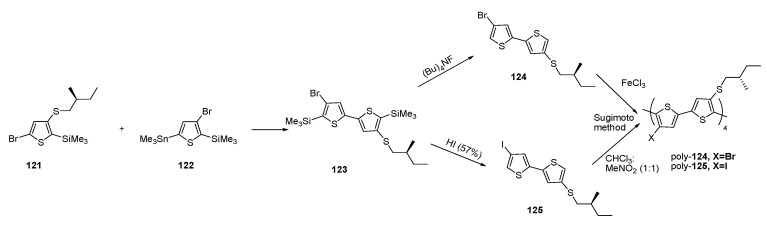
Synthesis of chiral unsymmetrically substituted bithiophenes serving as monomers for octithiophene analogues poly-**124** and poly-**125**.

Among the designed structures of chiral unsymmetrical *β*-alkylsulfanyl bithiophenes are 4-bromo-4′-[(*S*)-2-methylbutylsulfanyl]-2,2′-bithiophene **124** and 4-iodo-4′-[(*S*)-2-methylbutylsulfanyl]-2,2′-bithiophene **125**, which were synthesized as shown in Scheme 36. The Stille-type coupling between a tin- (**122**) and a halo-derivative (**121**) was applied to obtain the intermediate bithiophenes **123**. Tert-butylammonium fluoride was used for the desilylation to form compound **124**, while 58% hydroiodic acid was the desilylating agent and allowed the bromide to be replaced with iodide at once to give compound **125**.

## 5. Syntheses of Other Thiophenes Functionalized with a Substituent Containing a Stereogenic Carbon Atom or a Stereogenic Heteroatom

**Scheme 37:**
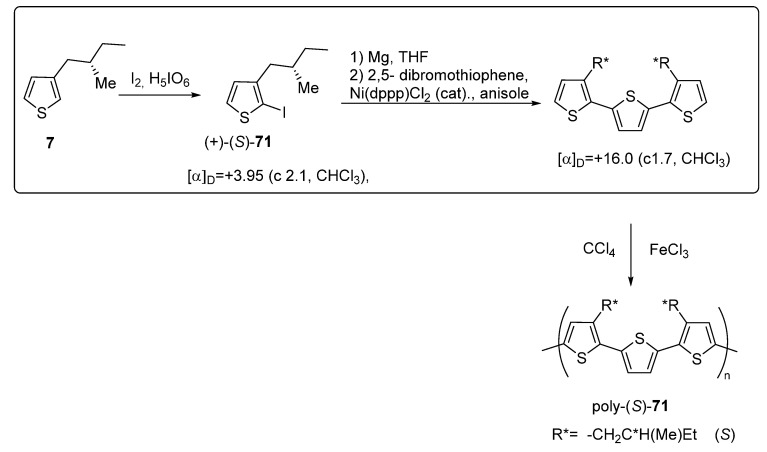
Synthesis of terthiophene derivative and its oxidative polymerization [39] following the general method of Sugimoto et al. using CCl_4_ as a solvent.

The transformations of the chiral monothiophene **7** into 2-iodo-3-[(S)-(+)-2-methylbutyl]thiophene (+)-(*S*)-**71** followed by the coupling with 2,5-dihalogenated thiophene provided 3,3′′-di[(*S*)-(+)-2-methylbutyl]-2,2′:5′,2′′-terthiophene, which served as a precursor to a new regioregular optically active polymer synthesized by FeCl_3_ oxidative coupling polymerization (Scheme 37) [39].

**Scheme 38:**
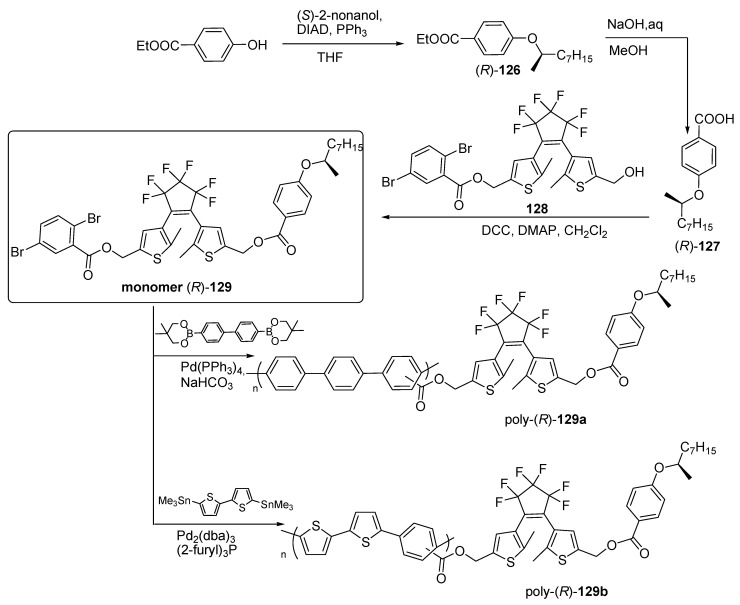
Synthesis of monomer (*R*)-**129** and the routes to the copolymerizations via the Suzuki or Stille coupling methods.

The synthesis of chiral (*R*)- and (*S*)-copolymers consisting of poly(*p*-terphenylene)s and poly(bithienylene-phenylene)s has been developed [99]. The polymers were distinguished by valuable fluorescent and photo responsive properties. Upon irradiation of ultraviolet and visible light, the polymers showed reversible quenching and emitting behaviors as a result of the photo-chemical isomerization of the dithienylethene fragments. The precursor of these polymers is the monomer (*R*)-**129**, the synthetic route of which is shown in Scheme 38.

The chiral fragment was delivered by a 2-methyloctyl chain appended to the hydroxyl of the 4-hydroxybenzoic acid. For its introduction, ethyl ester of the 4-hydroxybenzoic acid was coupled with (*S*)-2-nonanol under the Mitsunobu reaction conditions to obtain (*R*)-**126**.

The hydrolysis of ethyl-4-[(*R*)-1-methyloctoxy]benzoate (*R*)-**126** gave a free carboxylic function. The benzoic acid derivative (*R*)-**127** thus formed, reacted with the dithienylethene derivative **128** via coupling between the hydroxy group and the carboxyl group of (*R*)-**127**, which was carried out in the presence of dicyclohexylcarbodiimide (DCC) and 4-(dimethylamino)pyridine (DMAP), leading to the formation of the monomer (*R*)-**129**. The copolymerizations occurred through a Suzuki coupling reaction between (*R*)-**136** and 4,4′-biphenyldiboronic acid bis(propyl glycol)cyclic ester in the presence of Pd(PPh_3_)_4_ or through a Stille coupling between (*R*)-**129** and 5,5′-bis-trimethylstannyl-2,2′-bithiophene, catalyzed by Pd_2_(dba)_3_ (2-furyl)_3_P, to give poly-**129a** or poly-**129b**, respectively.

**Scheme 39:**
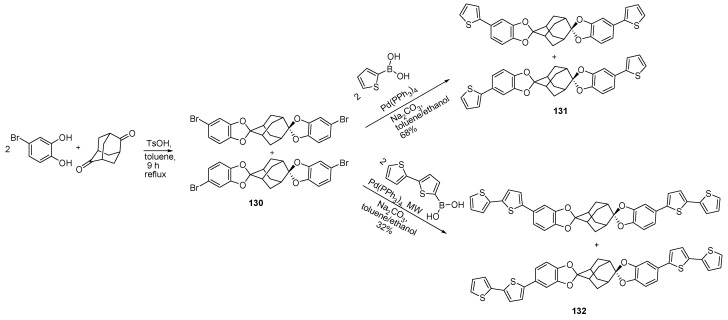
Synthesis of thienyl-based monomers **131** and **132** containing axially chiral adamantyl segments as their core.

The axial stereogenicity was introduced into the skeleton of a π-conjugated copolymer by a new molecular building block with versatile synthetic handles, a tetrasubstituted adamantane derivative **130**, synthesized from achiral 2,6-adamantanedione and 4-bromocatechol. Thus, the formed racemic bromide **130** was further converted into the electroactive monomers **131** and **132** after adopting the Suzuki coupling methodology (Scheme 39). The structures of the two monomers differed in terminal thienyl units, the first of those contained the monothiophenes at the ends of the core, while the second contained the terminal bithiophene units. The racemic electroactive polymers built from axially stereogenic adamantyl segments**,** were synthesized by electropolymerization of the monomers **131** and **132,** respectively [100].

## 6. Conclusions

In summary, this mini-review discusses the synthetic routes to mono- or bithiophene derivatives containing substituent(s) with a chiral element (stereogenic center or chiral axis) that can then serve as substrates in the polymerization reaction. We hope that the protocols for the synthesis of chiral monomeric thiophenes presented in this article will help practitioners of organic chemistry to design an appropriate pathway to obtain new valuable thiophene derivatives as chiral precursors of conjugated (co)polymers. The review covers the synthetic routes to derivatives that are functionalized at the β (β’) positions of the thiophene rings and those that are functionalized at one or both of the α positions. This division is due to the fact that, depending on the polymerization method under consideration, differently substituted mono- or bithiophenes are needed. The intended second part of the review will discuss various polymerization methods, the choice of which depends on the ease of access to the optically active monomeric substrate and the laboratory conditions allowing the selected polymerization technique to be used from among the following: McCullough’s approach, the Grignard metathesis (GRIM) reaction, the Rieke zinc coupling reaction, the Yamamoto condensation, the dehydrohalogenative polycondensation, the Kumada catalyst transfer condensation polymerization (KCTCP), the FeCl_3_-mediated polymerization (chemical oxidative polymerization), other transition metal catalyzed methods based on the Stille, Suzuki cross-coupling reactions or electropolymerization.

## Figures and Tables

**Table 1:** Transetherification reaction of 3,4-dimethoxythiophene **97** and (chiral) diols **98a**–**d** to (enantiomerically pure) disubstituted EDOTs **99a**–**d**.

	Glycol Deriv.	EDOT Deriv.	Yield [%]
1	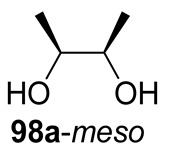	**99a**-*meso*	20
2	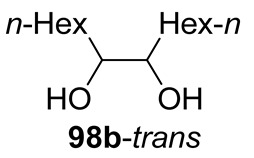	**99b**-*trans*	79
3	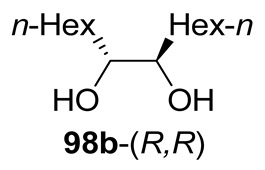	**99b**-(*R*,*R*)	73
4	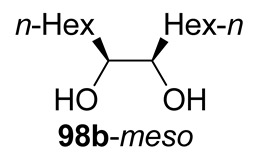	**99b**-*meso*	82
5	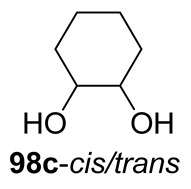	**99c**-*cis*/*trans*	63
6	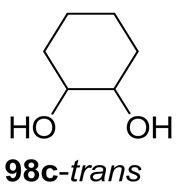	**99c**-trans	65
7	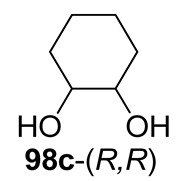	**99c**-(*R*,*R)*	68
8	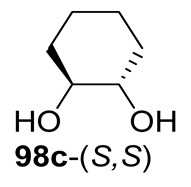	**99c**-(*S*,*S*)	66
9	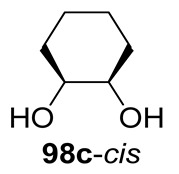	**99c**-*cis*	52
10	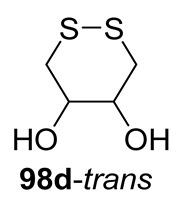	**99d**-*trans*	47
